# Demographic features of identified PLWHA infected through commercial and nonmarital noncommercial heterosexual contact in China from 2015 to 2018: a retrospective cross-sectional study

**DOI:** 10.1186/s12879-020-05757-2

**Published:** 2021-01-13

**Authors:** Zhilong Dong, Liying Ma, Chang Cai, George Fu Gao, Fan Lyu

**Affiliations:** 1 National Institute for Viral Disease Control and Prevention, Chinese Center for Disease Control and Prevention, 155 Changbai Road, Changping District, Beijing, 102206 China; 2 National Center for AIDS/STD Control and Prevention, Chinese Center for Disease Control and Prevention, 155 Changbai Road, Changping District, Beijing, 102206 China

**Keywords:** HIV/AIDS, Heterosexual transmission, Heterosexual contact, Commercial heterosexual contact, Nonmarital and noncommercial heterosexual contact

## Abstract

**Background:**

Understanding the demographic characteristics of people living with HIV/AIDS (PLWHA) infected through commercial heterosexual contact (CHC) or nonmarital noncommercial heterosexual contact (NMNCHC) is important for HIV/AIDS prevention and control.

**Methods:**

Cases reported through the Chinese HIV/AIDS Case Reporting System (CRS) from 2015 to 2018 were analyzed. A descriptive and preliminary inferential analysis were performed for those demographic characteristics deemed of interest.

**Results:**

Overall, 523,121 identified PLWHA between 2015 and 2018 in the CRS were analyzed. The constituent ratio of heterosexual transmission increased from 66.25% in 2015 to 71.48% in 2018. The proportion of CHC heterosexual transmission decreased from 40.18% in 2015 to 37.99% in 2018, while that of NMNCHC increased from 46.33% in 2015 to 49.02% in 2018. PLWHA infected through NMNCHC were significantly younger than those who were infected through CHC (Student’s t test, *P* < 0.0001), with an average age gap ranging from 5.63 (2015) to 7.46 (2018) years, and the average age of both groups increased annually. The frequency of newly identified PLWHA who were infected through CHC had a remarkable increase among the ages of 65 and above. Gender distribution was significantly different between CHC and NMNCHC (χ^2^ = 8909.00(2015), 9941.90(2016), 11,004.00 (2017), 12,836.00(2018), all *P* < 0.0001), and the ratio of men to women in the NMCHC group was 1.50:1 (2015), 1.51:1 (2016), 1.54:1 (2017), and 1.52:1 (2018), while in the commercial heterosexual contact (CHC) group, these ratios were 11.45:1 (2015), 12.08:1 (2016), 12.53:1 (2017), and 13.28:1 (2018). Marital status was significantly different between CHC and NMNCHC (χ^2^ = 94.67 (2015), 109.88(2016), 58.18(2017), 152.38(2018), all *P* < 0.0001). As the educational level improved, the proportion of NMNCHC also increased (Cochran - Armitage test, *P* < 0.0001).

**Conclusions:**

We found that heterosexual transmission was the primary mode of HIV transmission in China from 2015 to 2018. PLWHA infected through CHC and NMNCHC had different characteristics in age, gender, marital status, and educational level. The frequency of PLWHA infected through CHC increased substantially in the age group of 65 and above. This study provides useful baseline data for future studies on the heterosexual transmission of HIV in China.

## Background

In China, sexual transmission has already become the predominant route of human immunodeficiency virus (HIV) infection [1]. In 2013, 90.8% of newly identified HIV infections and acquired immune deficiency syndrome (AIDS) cases were reported to have been transmitted through sexual contact, among which heterosexual transmission accounted for 69.4% of the total cases [2]. During the following years, newly reported people living with HIV/AIDS (PLWHA) infected through heterosexual contact made up approximately two-thirds of the total number of cases [3, 4]. According to the report of the *National AIDS and STD Epidemi*c in the third quarter of 2018, 29,416 cases of newly identified HIV/AIDS cases in China were transmitted through heterosexual contact, accounting for 71.1% of the total [5].

Heterosexual transmission can be broadly divided into three types: those acquired within marriage or another type of live-in partnership, those acquired when selling or buying sex and those acquired in sex with a casual partner, including transient girlfriends and boyfriends [6]. Previous studies of heterosexual transmission have mainly focused on commercial heterosexual transmission and transmission within marriage [7, 8], and there are few studies that subdivided the modes of heterosexual transmission and conducted a synthesized analysis. To distinguish among different types of heterosexual contact, the option of a contact history, including commercial or noncommercial heterosexual contact, was added as a subclassification of nonmarital heterosexual transmission in the Chinese HIV/AIDS Case Reporting System (CRS) in 2014. Based on this, we were able to analyze the characteristics of PLWHA transmitted through commercial heterosexual contact (CHC) or nonmarital noncommercial heterosexual contact (NMNCHC).

Both CHC and NMNCHC are important components of heterosexual transmission. CHC plays a significant role in HIV transmission [9, 10], and female sex workers (FSWs) and male clients of FSWs are among the key populations monitored by the National Sentinel Surveillance System [11]. In 2015, the overall HIV positive rate of FSWs was 0.19%, while the rates were 0.39, 0.10 and 0.07% among FSWs from low-, medium- and high-level venues, respectively [12]. Despite a seemingly low HIV prevalence among FSWs in China, the scale and risk of HIV infection is enormous given the size of the population of FSWs and their clients [13]. Many studies have shown that the male clients of FSWs are important bridges for HIV transmission from FSWs to the low-risk general population [14–18]. In addition, several regional studies with comparatively small sample sizes noticed that NMNCHC accounted for an increasingly high proportion of nonmarital heterosexual transmission [19–24]. However, piecemeal data cannot provide a broad picture of nationwide epidemics. In the meantime, the concealment and diversity of NMNCHC made it difficult to understand its characteristics and significantly increased the difficulty of controlling the spread of HIV [22].

Since different transmission routes have varied characteristics and risk factors, the HIV/AIDS prevention and control strategies for heterosexual transmission should be formulated and carried out according to the different heterosexual transmission modes [25, 26]. Understanding the characteristics of cases infected through CHC and NMNCHC is important for HIV/AIDS prevention work in China. However, there is a lack of national-level analysis on the epidemic characteristics of those two transmission routes. Due to these considerations, this study applied the CRS data from 2015 to 2018 to synthetically describe and preliminarily compare the demographics of NMNCHC and CHC.

## Methods

### Data collection

China established the HIV/AIDS case reporting system in 1985. HIV blood testing includes initial screening and confirmation testing based on the National Guideline for the Detection of HIV/AIDS. An individual who tests positive on the confirmatory test is determined to be HIV-infected [27]. Newly identified cases of HIV infection are reported through this web-based system by individuals from the local Centers for Disease Control and Prevention (CDC) and medical institutions [28]. Completed case reports are then communicated to the National Center for AIDS/STD Control and Prevention (NCAIDS) for data quality monitoring and logic checks. Local CDC personnel and NCAIDS staff double evaluate and identify mistakes in logic and duplication at the local and national level. These measures ensure that the system can obtain accurate information on PLWHA throughout the country [29].

### Data management

The use of data from the CRS for this study was authorized by the National Center for AIDS/STD Control and Prevention, China CDC. All identified PLWHA (523,121 cases) captured from 2015 to 2018 in the CRS were included in our study. Specific inclusion and exclusion criteria were developed to meet the objectives of this study. The inclusion criterion was cases reported between 2015 and 2018. Cases that did not know the date of birth, lacked key information, or had faulty logic were excluded. Personal information was removed from the database prior to analysis to protect the participants’ privacy. Demographic factors, including age, gender, marital status, and educational level, were included in the analysis [28].

### Definitions

a. Nonmarital heterosexual contact (NMHC): sexual contact with heterosexual partners who were not married, including commercial and noncommercial heterosexual contact. Those who contracted HIV within marriage or from another type of live-in partnership were excluded.

b. Commercial heterosexual contact (CHC): commercial sexual contact with a nonmarital heterosexual partner, including those that contracted HIV when selling or buying sex.

c. Nonmarital noncommercial heterosexual contact (NMNCHC): noncommercial sexual contact with a heterosexual partner who was not married, including those that acquired HIV from transient girlfriends and boyfriends, as well as from any other casual heterosexual partners.

d. “HIV” refers to the presence of HIV infection at the time of reporting, “AIDS” refers to diagnosed AIDS patients, and “HIV/AIDS” refers either to an HIV infection or an AIDS case [26].

### Statistical analysis

Descriptive analysis: demographic characteristics such as routes of transmission, gender, age, marital status and educational level were taken into consideration. The compositions of all transmission routes, as well as heterosexual transmission were explored. In the meantime, the frequency and constituent ratio of CHC and NMNCHC were analyzed by gender. The average age, as well as the frequencies and proportions (%) of all categorical factors (gender, marital status, and educational level) and their subgroups, were calculated for the successive study years.

Inferential analysis: the differences in the mean age of the CHC and NMNCHC groups and the differences in the mean age by gender of each year were analyzed. The distribution differences in gender, marital and educational status were compared between the two groups. The means of age were compared using the Student’s t-test, the distribution differences were analyzed using the Chi-square test, and trends were tested using the Cochran-Armitage test. Values of *P* < 0.05 were considered statistically significant.

Statistical software: data analysis was performed using SPSS 21.0 software (IBM Inc., Armonk, NY, USA), R (The R Foundation for Statistical Computing, R 3.6.1), RStudio interface (RStudio, Inc. Version 1.2.5033), and Microsoft Excel 2019 (Microsoft Corp 2019).

## Results

### Description analysis

**Fig. 1:**
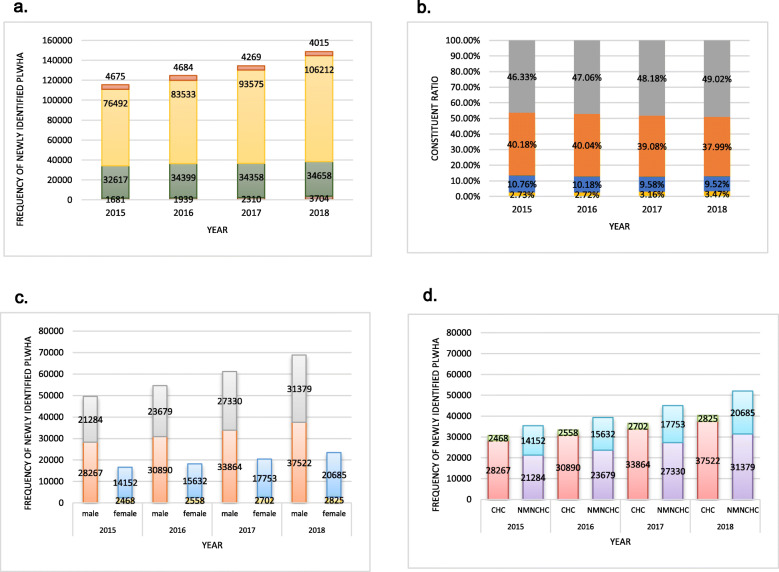
**a** Frequency of newly identified PLWHA of diverse HIV transmission routes from 2015 to 2018. **b** Constituent ratio of various heterosexual transmission routes from 2015 to 2018. **c** Frequency of newly identified PLWHA infected through CHC and NMNCHC in males and females from 2015 to 2018. d: Frequency of newly identified PLWHA by gender among those infected through CHC and NMNCHC from 2015 to 2018. *CHC: commercial heterosexual contact; NMNCHC: nonmarital noncommercial heterosexual contact; MC: marital contact; NC: non-classified; PLWHA: people living with HIV/AIDS. a  . b  . c  . d

Overall, 523,121 identified HIV/AIDS cases captured from 2015 to 2018 in the CRS were analyzed, among which 359,812 (68.78%) cases were reported as having been contracted through heterosexual contact (HC). The proportion of people infected through HC increased yearly from 66.25% (2015) to 71.48% (2018) (Fig. 1a). Among all heterosexual transmissions, the proportion of CHC decreased from 40.18% (2015) to 37.99% (2018), whereas the proportion of NMNCHC increased from 46.33% (2015) to 49.02% (2018) (Fig. 1b). Among the men who reported transmission through nonmarital heterosexual contact, the proportion of the CHC group declined year by year, from 57.05% (2015) to 54.46% (2018), while the proportion of NMNCHC increased from 42.95% (2015) to 45.54% (2018). In the female nonmarital heterosexual group, the proportion of the CHC group decreased from 14.85% (2015) to 12.02% (2018), while the NMNCHC proportion was much higher and increased annually from 85.15% (2015) to 87.98% (2018) during the years that the study focused on (Fig. 1c). In the CHC group, the proportion of men was quite high and increased yearly from 91.97% (2015) to 93.00% (2018). Correspondingly, the female proportion decreased from 8.03% (2015) to 7.00% (2018). The gender ratios (male: female) were 11.45:1, 12.08:1, 12.53:1, and 13.28:1 from 2015 to 2018, respectively. In the NMNCHC group on the other hand, the gender proportion was relatively stable, with males accounting for 60.06 to 60.27%, and the gender ratios (male: female) being 1.50:1, 1.51:1, 1.54:1, and 1.52:1 from 2015 to 2018, respectively (Fig. 1d).

During the years analyzed in this study, the average age of the two groups increased annually. The average age of the CHC group increased from 48.32 (2015) to 52.69 (2018), and the average age of the NMNCHC group increased from 42.69 (2015) to 45.23 (2018). Meanwhile, the married proportion of the CHC group was relatively stable, ranging from 51.20% in 2015 to 51.48% in 2018, while the married proportion of the NMNCHC increased from 49.10% in 2015 to 51.02% in 2018. The proportion of divorced or widowed individuals increased annually, while the percentage of those who were unmarried decreased in both groups. The educational level in the CHC group changed during the study years; the proportion of those having graduated junior high school and above decreased annually, while the proportion of those only having graduated primary school as well as those who were illiterate increased annually. The NMNCHC group followed a similar trend, except that the proportion of individuals educated in college or above was comparatively stable, only reducing by 0.68% (from 10.10% in 2015 to 9.52% in 2018), compared with a 2.05% (from 7.39% in 2015 to 5.34% in 2018) decline in the CHC group (Table 1).

**Table 1:** Demographics of newly identified PLWHA infected through CHC and NMNCHC from 2015 to 2018

		2015	2016	2017	2018
**CHC**	**Average Age**	48.32	49.95	51.03	52.69
	**Gender**				
	Male	28,267 (91.97%)	30,890 (92.35%)	33,864 (92.61%)	37,522 (93.00%)
	Female	2468 (8.03%)	2558 (7.65%)	2702 (7.39%)	2825 (7.00%)
	**Marital status**				
	Married	15,735 (51.20%)	17,274 (51.64%)	19,084 (52.19%)	20,772 (51.48%)
	Divorced or widowed	8495 (27.64%)	9440 (28.22%)	10,430 (28.52%)	12,020 (29.79%)
	Unmarried	6384 (20.77%)	6620 (19.79%)	6898 (18.86%)	7453 (18.47%)
	Unknown	121 (0.39%)	114 (0.34%)	154 (0.42%)	102 (0.25%)
	**Educational level**				
	College and above	2271 (7.39%)	2158 (6.45%)	2167 (5.93%)	2153 (5.34%)
	High school or technical Secondary school	3778 (12.29%)	3779 (11.30%)	3788 (10.36%)	3901 (9.67%)
	Junior high school	11,474 (37.33%)	12,226 (36.55%)	12,808 (35.03%)	13,058 (32.36%)
	Primary school	10,915 (35.51%)	12,499 (37.37%)	14,459 (39.54%)	17,054 (42.27%)
	Illiteracy	2297 (7.47%)	2786 (8.33%)	3344 (9.15%)	4180 (10.36%)
	Subtotal	**30,735**	**33,448**	**36,566**	**40,347**
**NMNCHC**	**Average Age**	42.69	43.42	44.47	45.23
	**Gender**				
	Male	21,284 (60.06%)	23,679 (60.24%)	27,330 (60.62%)	31,379 (60.27%)
	Female	14,152 (39.94%)	15,632 (39.76%)	17,753 (39.38%)	20,685 (39.73%)
	**Marital status**				
	Married	17,400 (49.10%)	20,025 (50.94%)	23,059 (51.15%)	26,562 (51.02%)
	Divorced or widowed	9554 (26.96%)	10,215 (25.99%)	12,368 (27.43%)	14,351 (27.56%)
	Unmarried	8221 (23.20%)	8885 (22.60%)	9460 (20.98%)	10,869 (20.88%)
	Unknown	261 (0.74%)	186 (0.47%)	196 (0.43%)	282 (0.54%)
	**Educational level**				
	College and above	3578 (10.10%)	4121 (10.48%)	4483 (9.94%)	4904 (9.42%)
	High school or technical Secondary school	4971 (14.03%)	5479 (13.94%)	5939 (13.17%)	6227 (11.96%)
	Junior high school	12,871 (36.32%)	13,721 (34.90%)	15,620 (34.65%)	16,270 (31.25%)
	Primary school	10,605 (29.93%)	12,090 (30.75%)	14,116 (31.31%)	17,166 (32.97%)
	Illiteracy	3411 (9.63%)	3900 (9.92%)	4925 (10.92%)	7497 (14.40%)
	**Subtotal**	**35,436**	**39,311**	**45,083**	**52,064**
	**Total**	**66,171**	**72,759**	**81,649**	**92,411**

*Abbreviation*: *CHC* Commercial heterosexual contact, *NMNCHC* Nonmarital noncommercial heterosexual contact, *PLWHA* People living with HIV/AIDS

**Fig. 2:**
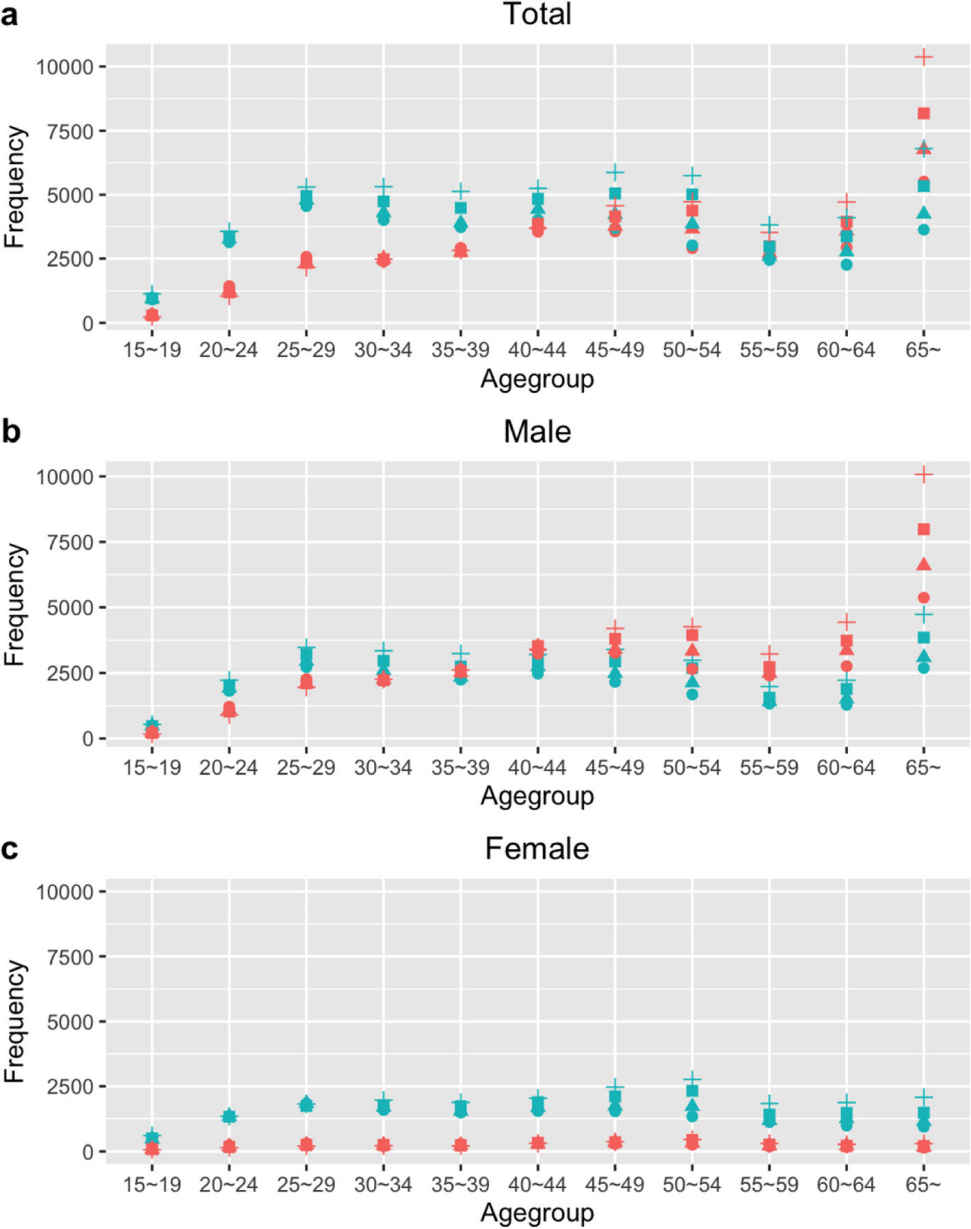
**a** Frequency of newly identified PLWHA infected through CHC and NMNCHC in different age groups from 2015 to 2018. **b** Frequency of newly identified PLWHA infected through CHC and NMNCHC in different age groups from 2015 to 2018 among males. **c** Frequency of newly identified PLWHA infected through CHC and NMNCHC in different age groups from 2015 to 2018 among females. *CHC: commercial heterosexual contact; NMNCHC: nonmarital noncommercial heterosexual contact; PLWHA: people living with HIV/AIDS

During the period in question, the frequency of transmission in the NMNCHC group was higher than that of the CHC group in all age groups under 55 years old, especially in the age group from 20 to 40. However, there was an obvious difference in the age group over 60, in which the number of transmissions in the NMNCHC group was much lower than that of the CHC group. This difference peaked in the age group over 65, in which the frequency of identified PLWHA in the CHC group was 1.52 to 1.59 times that of the NMNCHC group. In the 65+ age group, the CHC group showed a substantial increase, and the proportion of cases identified in the over 65 years in the CHC group increased from 17.92% (2015) to 25.71% (2018), while that in the NMNCHC group increased from 10.24% (2015) to 13.06% (2018) (Fig. 2a).

In the CHC group, the 60 to 64 as well as 65 and above age groups had much higher sex ratios (male: female) than the total ratios, which were 15.34:1 (2015), 14.94:1 (2016), 18.06:1 (2017), and 16.64:1 (2018) in the 60 to 64 age group and 37.80:1 (2015), 38.33:1 (2016), 40.12:1 (2017), and 34.16:1 (2018) in the 65 and above age group. Under the age of 40, most age groups of CHC showed a downward trend, but after age 40, there was an increase that peaked at 65+ for both genders. Women made up a small proportion in all age groups, hence the increase in CHC was mainly driven by men in the older age groups (Fig. 2b, c). On the other hand, in the NMNCHC group, among the age group of 65~, the sex ratios (male: female) were 2.87:1 (2015), 2.71:1 (2016), 2.61:1 (2017), and 2.28:1 (2018). The sex ratio remained relatively stable in all age groups in the NMNCHC; however, there was an obvious increase in the younger age groups, especially in the age group of 25 to 29, which rose from 1.49:1 to 1.91:1 during the years analyzed in this study and became the second highest among all age groups in 2018 (Fig. 2b, c).

### Inferential analysis

There was a significant age difference between PLWHA infected through CHC and those infected through NMNCHC from 2015 to 2018 (*P* < 0.0001). Those who were infected through NMNCHC were significantly younger than those cases transmitted through CHC (*P* < 0.0001), with an average age gap ranging from 5.63 to 7.46 years. The age of male PLWHA in the CHC group was significantly higher than that in the NMNCHC group (*P* < 0.0001), and the mean age difference was 5.46 years (2015) to 7.82 years (2018). In the female group, there was no significant difference in age between the two groups in 2015 (*P* = 0.398). The age among women of the CHC cases was significantly higher than that of NMNCHC from 2016 to 2018 (2016: *P* < 0.0001, 2017: *P* = 0.029, 2018: *P* < 0.0001), but the age differences were much smaller than those of men, ranging from only 0.27 (2015) to 1.62 (2018) (Table 2).

**Table 2:** Overall age differences and age differences by gender between newly identified PLWHA infected through CHC and NMNCHC from 2015 to 2018

Gender	Year	Contact History	Average Age by Year	N	SD	T (*Student’s t test*)	P	Age Gap
Both Male and Female	2015	**CHC**	48.32	30,735	15.79	46.36	*P* < 0.0001	5.63
		**NMNCHC**	42.69	35,436	15.39			
	2016	**CHC**	49.95	33,448	15.7	56.416	*P* < 0.0001	6.53
		**NMNCHC**	43.42	39,311	15.44			
	2017	**CHC**	51.03	36,566	15.61	59.88	*P* < 0.0001	6.56
		**NMNCHC**	44.47	45,083	15.53			
	2018	**CHC**	52.69	40,347	15.5	72.145	*P* < 0.0001	7.46
		**NMNCHC**	45.23	52,064	15.64			
Male	2015	**CHC**	48.88	28,267	15.8	37.97	*P* < 0.0001	5.46
		**NMNCHC**	43.42	21,284	15.91			
	2016	**CHC**	50.45	30,890	15.72	47.71	*P* < 0.0001	6.52
		**NMNCHC**	43.94	23,679	15.94			
	2017	**CHC**	51.54	33,864	15.63	52.31	*P* < 0.0001	6.74
		**NMNCHC**	44.80	27,330	16.11			
	2018	**CHC**	53.13	37,522	15.5	64.66	*P* < 0.0001	7.82
		**NMNCHC**	45.31	31,379	16.19			
Female	2015	**CHC**	41.85	2468	14.14	0.85	*P* = 0.398	0.27
		**NMNCHC**	41.59	14,152	14.51			
	2016	**CHC**	43.84	2558	14.08	3.9	*P* < 0.0001	1.21
		**NMNCHC**	42.63	15,632	14.63			
	2017	**CHC**	44.61	2702	13.92	2.18	*P* = 0.029	0.65
		**NMNCHC**	43.96	17,753	14.59			
	2018	**CHC**	46.73	2825	14.18	5.58	*P* < 0.0001	1.62
		**NMNCHC**	45.11	20,685	14.77			

*Abbreviation: CHC* Commercial heterosexual contact, *NMNCHC* Nonmarital noncommercial heterosexual contact, *PLWHA* People living with HIV/AIDS

Chi-square analysis of gender distribution of PLWHA infected through CHC and NMNCHC showed significant differences, χ^2^ (2015) =8909.00, *P* < 0.0001, χ^2^ (2016) =9941.90, *P* < 0.0001, χ^2^ (2017) =11,004.00, *P* < 0.0001, χ^2^ (2018) =12,836.00, *P* < 0.0001. In the meantime, the Chi-square tests of marital status between the two groups revealed that the distribution of marital status was significantly different between the groups in all years of the study, χ^2^ (2015) =94.67, *P* < 0.0001, χ^2^ (2016) =109.88, *P* < 0.0001, χ^2^ (2017) =58.18, *P* < 0.0001, χ^2^ (2018) =152.38, *P* < 0.0001. The distribution trends of CHC and NMNCHC for each year were tested by educational level, and Z (2015) = 10.22, *P* < 0.0001, Z (2016) =17.88, *P* < 0.0001, Z (2017) =20.78, *P* < 0.0001, Z (2018) =16.34, *P* < 0.0001, which indicated that the proportion of NMNCHC increased with the improvement in educational level (Table 3).

**Table 3:** Comparison of gender, marital status, and educational level between newly identified PLWHA infected through CHC and NMNCHC from 2015 to 2018

	2015		2016		2017		2018	
	χ^2^	P	χ^2^	P	χ^2^	P	χ^2^	P
**Gender** **(***Chi square test***)**	8909.00	*P* < 0.0001	9941.90	*P* < 0.0001	11,004.00	*P* < 0.0001	12,836.00	*P* < 0.0001
**Marital status** **(***Chi square test***)**	94.67	*P* < 0.0001	109.88	*P* < 0.0001	58.18	*P* < 0.0001	152.38	*P* < 0.0001
	Z	P	Z	P	Z	P	Z	P
**Educational level** **(***Cochran-Armitage test***)**	10.22	*P* < 0.0001	17.88	*P* < 0.0001	20.78	*P* < 0.0001	16.34	*P* < 0.0001

*Abbreviation*: *CHC* Commercial heterosexual contact, *NMNCHC* Nonmarital noncommercial heterosexual contact, *PLWHA* People living with HIV/AIDS

## Discussion

To our knowledge, this is the first study to analyze and compare the basic demographic characteristics of PLWHA reported as transmitted through CHC and NMNCHC at the national level. By analyzing data from the Chinese HIV/AIDS CRS from 2015 to 2018, we have gained a preliminary understanding of the demographics and differences between CHC and NMNCHC. This study provides further indications that heterosexual contact has become the main mode of HIV transmission in China. Furthermore, the proportion of NMNCHC in heterosexual transmission increases annually, indicating that NMNCHC is playing an increasingly important role. These findings support the conclusions of some small regional studies at the national level [22–24].

In nonmarital transmissions, men were more likely to be reported as transmitted through CHC, while the opposite was true for women. Furthermore, the ratio of males to females was much higher in the CHC group than in the NMNCHC group. This significant difference in the male-female ratio between CHC and NMNCHC indicates that men are more likely to be infected through CHC, while women are more likely to be infected through NMNCHC, but such a large difference in the proportion requires further study of the underlying causes. To explore the differences in the distribution and changing status of PLWHA frequency between CHC and NMNCHC within various age periods, we analyzed the frequency distribution over the years by age group. It was found that the two transmission routes displayed a much larger difference in some age groups, which indicated that age plays a crucial role in heterosexual transmission routes and should be fully considered when establishing prevention and control strategies.

In general, the average age of the CHC group was much higher than that of the NMNCHC group, which was mainly due to the much higher number of those infected through CHC over the age of 60, as well as the much lower frequency in cases under 60. In the meantime, the age difference between the two groups was mainly due to men. This discovery supports some other study findings that there is an increasing rate of HIV infection among older people [30–32], and that they were playing a more important role in local transmission [33]. Studies have found that many older Chinese adults remain sexually active [34, 35], and erectile dysfunction medications (EDMs) have become popular among older men to enhance sexual desire and performance [36]. Quite a number of older men choose low-end FSWs to satisfy their sexual needs [31], and due to the higher HIV prevalence among low-end FSWs [37], older adults’ lack of HIV/AIDS-related knowledge [34], and their unwillingness to use condoms during commercial sexual behavior [38], infection among older adults has soared in recent years. Generally, studies targeted HIV epidemics among older adults mainly focused on the population aged ≥50 years [31, 33]. However, our study found that the number of CHC transmission reports among people over the age of 65 has increased significantly. Therefore, it may be necessary to conduct specific studies on this age group in future studies.

Compared with CHC, younger adults are more likely to be infected with HIV through NMNCHC. Our study also noticed that the sex ratio in the 25–29 age group of NMNCHC transmissions had increased significantly. This may be because men aged 25 to 29 are relatively more active sexually and more likely to have casual sex and concurrent relationships. One study explored casual sexual behaviors among 108 Yi villagers aged 15–35 years from Liangshan Prefecture in Sichuan Province and found that 66.7% of them reported multiple sexual partnerships. Only 21.3% reported ever having used a condom, and 56.9% were involved in concurrent sexual partnerships among network members in components of size ≥3 [39]. In other parts of China, the transmission of HIV via NMNCHC in this age group needs further study.

The marital status was significantly different between the groups in all of the years studied, something that is probably because unmarried people are more likely to be younger and engaged in a casual heterosexual relationship, while divorced or widowed people are more likely to be older and to be engaged in commercial heterosexual behavior. Our study also discovered that the proportion of married PLWHA was increasing among those reported as infected through NMNCHC. This may suggest that marriage is becoming less of a barrier to extramarital sexual behavior in China. Furthermore, as the educational level improved, the proportion of NMNCHC also increased. This is probably because the educational level may have an association with socioeconomic status, which may affect the way people seek sexual partners. Age might also play an important role in the difference of educational levels between the two groups. Further studies are needed to rule out the effects of confounders and to explore these factors’ influence on the likelihood of being infected through CHC or NMNCHC.

Given the significant increase in the number of reports of transmission through CHC among persons over the age of 65, further study of this population is necessary in order to understand the key determinants of HIV transmission among them. At the same time, the investigation into this group is likely to reveal those FSWs who have transmitted HIV to these elderly men, which is conducive to strengthening the management of low-end FSWs and achieving prevention and control of HIV transmission. Moreover, because older adults are normally less educated, we need to adopt more understandable ways for publicity and education [40]. These could be simple methods such as using pictures as well as simple and clear language in order to impart knowledge on AIDS prevention and control, thereby improving the awareness of HIV/AIDS and increasing the condom utilization rate in this group. Moreover, the increase in NMNCHC transmission in the younger age groups is more difficult to control than CHC transmission. NMNCHC transmission networks are likely to be more widespread and complex, and current research on transmission in this population is very limited. There is a need for further study to understand transmission networks and the new ways in which younger adults acquire sexual partners, so as to establish new preventative measures in response to these new modes. In addition, this study preliminarily explored the different characteristics of CHC and NMNCHC. However, due to the regional differences in the HIV epidemic in China, the specific epidemic characteristics of each region need to be investigated separately.

### Limitations

There are several limitations to this study. The sexual contact history in the CRS is self-reported, and objective measures for verification are difficult to implement. Therefore, it is possible that the identified PLWHA lied about their contact history, and there could also be differential reporting between CHC and NMNCHC groups. Some HIV/AIDS patients may conceal their exposure to other high-risk sexual behaviors, such as a male’s exposure to homosexual sex or a female’s exposure to commercial sex [21]. Furthermore, “newly reported cases” should not replace the incidence as they may represent transmissions that occurred many years earlier [41]. Since the analysis is mainly conducted at the univariate or bivariate level, there may be confounding factors, and those potential factors related to CHC and NMNCHC need to be further explored. Moreover, there may be some unreported cases that could influence our conclusions. However, the large nationwide sample size might, to some extent, make up for these deficiencies.

## Conclusions

In summary, this is the first nationwide targeted analysis of the epidemic status of PLWHA infected through commercial and noncommercial contact in nonmarital heterosexual transmission. PLWHA infected through CHC and NMNCHC have different characteristics, such as age and gender, marital status and educational level. PLWHA reported as transmitted through CHC was increasingly more common among men aged 65 and above, and further research is needed to explore the reason for the rapid increase in newly identified PLWHA among this age group. NMNCHC played an increasingly significant role in heterosexual transmission, and PLWHA infected through NMNCHC were comparatively younger than those transmitted through CHC. Further study is necessary to understand the transmission characteristics of NMNCHC, such as the transmission networks and the new ways in which people obtain sexual partners. Our study provided useful basic data for the further exploration of the characteristics of the heterosexual transmission of HIV in China.

## Data Availability

The datasets used and analyzed during the current study are available from the corresponding author on reasonable request.

## Abbreviations

AIDS: Acquired immune deficiency syndrome
CHC: Commercial heterosexual contact
CRS: Case reporting system
FSW: Female sex workers
HC: Heterosexual contact
HIV: Human immunodeficiency virus
NMHC: Nonmarital heterosexual contact
NMNCHC: Nonmarital noncommercial heterosexual contact
PLWHA: People living with HIV/AIDS
